# Acute Pancreatitis as a First Presentation of Granulomatosis With Polyangiitis

**DOI:** 10.14309/crj.0000000000000986

**Published:** 2023-02-10

**Authors:** Michael Youssef, Mary Sedarous, Andrea Grin, Andrew Chung, Lawrence Hookey

**Affiliations:** 1 Division of Internal Medicine, University of Toronto, Toronto, Ontario, Canada; 2 Division of Gastroenterology, Kingston Health Sciences Centre, Queen's University, Kingston General Hospital, Kingston, Ontario, Canada; 3 Department of Pathology and Molecular Medicine, Kingston Health Sciences Centre, Queen's University, Kingston, Ontario, Canada; 4 Department of Radiology, Kingston Health Sciences Centre, Queen's University, Kingston, Ontario, Canada

**Keywords:** acute pancreatitis, GPA, vasculitis, anti-PR3:, endoscopic ultrasound, recurrent acute pancreatitis, endoscopic ultrasound, granulomatosis with polyangiitis, gastrointestinal manifestations of pancreatitis

## Abstract

Granulomatosis with polyangiitis (GPA) is a rare necrotizing antineutrophil cytoplasmic antibody-associated vasculitis characterized by inflammation in small-sized arteries. Gastrointestinal involvement is exceedingly rare in GPA. Here, we present a case of recurrent acute pancreatitis as the initial presentation of GPA. The diagnosis was made based on radiological and pathological findings of acute pancreatitis in conjunction with positive anti-PR3 antibody which is strongly associated with GPA. Systemic vasculitides are rare but important to consider in cases of idiopathic acute pancreatitis. Early diagnosis and therapy allow for high rates of remission and improved survival rates.

## INTRODUCTION

Granulomatosis with polyangiitis (GPA) is a rare necrotizing antineutrophil cytoplasmic antibody-associated vasculitis characterized by inflammation in small-sized arteries. GPA often presents with nonspecific constitutional symptoms and commonly involves a triad of (i) symptoms from the upper (nasal obstruction, sinusitis, and crusting rhinitis) and lower respiratory tract (lung nodules and alveolar hemorrhage), (ii) systemic vasculitis, and (iii) kidney involvement (necrotizing glomerulonephritis).^1,2^ However, gastrointestinal involvement is exceedingly rare and only occurs in about 5%–11% of GPA cases.^3^ Specifically, recurrent acute pancreatitis is even more uncommon.

## CASE REPORT

**Figure 1.**
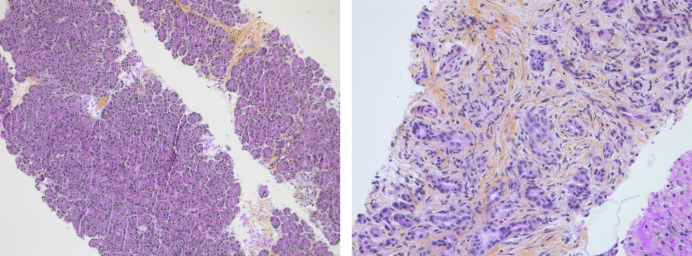
Pathology of pancreas showing focal chronic pancreatitis with fibrosis and mild lymphocytic infiltrate.

**Figure 2.**
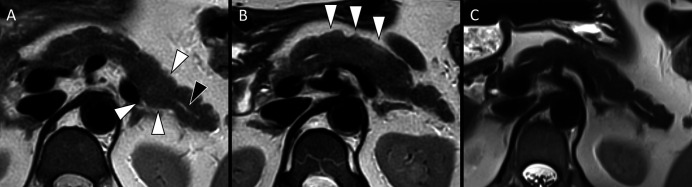
Serial T2-weighted magnetic resonance images of the pancreas. (A) Initial MRI (February 2021) demonstrates focal enlargement and T2 signal hyperintensity at the junction of the pancreatic body and tail (white arrowheads) with prominence of the upstream pancreatic duct (black arrowhead) suspicious for focal pancreatitis. (B) Follow-up MRI (March 2021) demonstrates worsening enlargement and signal abnormality of the pancreas, more diffusely involving the pancreatic body (white arrowheads). (C) Follow-up MRI (October 2021) demonstrates resolution of parenchymal changes with normal size and lobulated contour of the pancreas. MRI, magnetic resonance imaging.

A 48-year-old woman was seen in an outpatient gastroenterology clinic for recurrent idiopathic pancreatitis. She reported a 6-month history of intermittent sharp epigastric pain radiating to the back not associated with meals. This abdominal pain was associated with a rise in lipase. These symptoms were not associated with diarrhea or other changes in bowel habits. The patient noted having episodes of acute sinusitis shortly before the onset of her epigastric pain. Her initial laboratory work revealed normal creatinine 79, normal liver enzymes, elevated total bilirubin 24, lipase 316, mildly high C-reactive protein 16.1, and erythrocyte sedimentation rate 27 which normalized on repeat blood work. Abdominal ultrasound was unremarkable with no gallstones and intrahepatic or extrahepatic duct dilatation. An abdominal magnetic resonance imaging (MRI) revealed segmental enlargement of the distal tail/body of the pancreas consistent with resolving focal pancreatitis (Figure 1). A repeat abdominal MRI 5 weeks later revealed progression of pancreatic swelling and signal abnormality with intermittent narrowing of the pancreatic duct (Figure 1). These findings were suspicious for an inflammatory pancreatic process such as autoimmune pancreatitis. The patient then underwent an endoscopic ultrasound-guided examination and fine-needle aspirate of her pancreas. Of note, the papilla appeared normal endoscopically. Pathology revealed focal chronic pancreatitis with fibrosis and mild lymphocytic infiltrate, which was not consistent with autoimmune pancreatitis (Figure 2). She was then treated empirically with a 3-month course of prednisone 40 mg daily with a taper and had significant improvement in her symptoms as well as interval resolution of pancreatic inflammation on repeat MRI (Figure 1). After discontinuation of her steroids, her symptoms recurred with intermittent epigastric pain, facial pain, and sinusitis. She was then seen by rheumatology, and an autoimmune panel revealed positive anti-PR3 (27 RU/mL), negative anti-myeloperoxidase, and normal immunoglobulin G4 levels. Ultimately, a diagnosis of limited GPA, which spares the kidneys, was made, given the patient's clinical presentation and positive anti-PR3 antibody. Given that there was no life-threatening organ involvement, she was started on methotrexate therapy and had significant improvement in her symptoms. The plan is to stay on methotrexate for at least 24 months and have regular follow-up to ensure clinical stability. To our knowledge, this is the first case report on acute pancreatitis as the initial presentation of limited GPA.

## DISCUSSION

Gastrointestinal tract involvement is a rather rare entity in systemic vasculitides, especially GPA, and is often associated with poor prognosis.^4^ In a case series of 62 patients with vasculitis, gastrointestinal involvement was identified in only 5%–11% of GPA cases, compared with 30%–56% in microscopic polyangiitis and 40%–60% in polyarteritis nodosa.^3^ Pancreatic disease is even rarer; in fact, only 3 patients in that series had acute pancreatitis, and none of them had GPA.^3^ This certainly suggests that pancreatic involvement is exceedingly rare in GPA and even rarer as the initial presentation of the disease.^5^ Given this rare association, vasculitis is often overlooked in patients with idiopathic acute pancreatitis.^6^ As such, the diagnosis is often delayed as patients develop other manifestations of the disease or even until they present with life-threatening end-organ damage.^7^ This highlights the importance of having a high clinical suspicion for systemic vasculitis in cases of idiopathic pancreatitis and the important role of collaborative multidisciplinary involvement in multisystemic patient presentations. This allows for early recognition, timely treatment, and best possible outcomes for the patient.

It is interesting to note that our patient had an atypical presentation for GPA because there was no evidence of any respiratory or renal involvement clinically or radiologically. This was most consistent with a diagnosis of limited GPA, which is uncommon but reported in previous case reports where there was pulmonary and/or renal sparing.^8^ This makes our case unique firstly, given the diagnosis of limited GPA which is different from other GPA pancreatitis case reports where patients had other organ involvement simultaneously (Table 1). However, it is also possible that our patient was diagnosed and treated before disease progression to involve other organ systems. Second, our patient had always been relatively stable, with only brief hospital admissions for pancreatitis, and did not require any acute treatments for her vasculitis. Besides an empiric trial of short steroid course, she did not require any pulse methylprednisolone or induction immunosuppressive therapies (eg, cyclophosphamide or rituximab). This is in contrast to previous literature where gastrointestinal involvement predicted more severe disease, often with very poor outcomes despite aggressive treatment.^9,10^ Nevertheless, it is important to continue close observation and have a low threshold for multiorgan screening while our patient is maintained on methotrexate therapy.

**Table 1.** Literature review of previous case reports on acute pancreatitis as initial presentation of GPA

Study Patient	Initial presentation	Laboratory work	Imaging	Pathology	Hospital course	Treatment	Outcome
Tyagi et al^6^ 43/M	Acute pancreatitis	Leukocytosis, elevated lipase 495, amylase 362, positive c-ANCA and anti-PR3 levels	CT abdomen: diffusely edematous pancreas with normal bile duct CT chest: nodular lesions/alveolar hemorrhages	N/A	Palpable purpura, CT chest shows nodular lesions and alveolar hemorrhage	Steroids, cyclophosphamide, azathioprine (maintenance)	Clinically stable at 9-months follow-up
Tao et al^13^ 66/F	Acute pancreatitis	Elevated lipase 388, amylase 96, normal LFTs, CRP 166, positive c-ANCA and anti-PR3 levels, IgG4 1.16, mild CA19-9 elevation, negative anti-GBM, negative MPO-ANCA	CT abdomen: focal pancreatitis with multifocal hypodense lesions in the pancreatic head and tail MRCP: edematous swollen pancreas with small pancreatic collections in keeping with pancreatitis	ERCP-based biopsy of pancreas showed no malignancy but presence of chronic inflammation with some IgG4+ plasmacytes (<30%) without fibrosis or eosinophilia	CT chest showed multiple focal nodular parenchymal lung lesions that were believed to be secondary to a systemic vasculitis	Steroids, cyclophosphamide	Complete resolution of symptoms after 2 wk
Abu-Hilal et al^14^ 20/F	Acute pancreatitis	Leukocytosis, elevated CRP 145, normal amylase 20, positive c-ANCA with high anti-PR3 levels >600, negative anti-GBM and MPO-ANCA	CT abdomen: edematous pancreatic tail, with thrombosed splenic vein and a markedly abnormal spleen	Small and large bowel histology showed ischemic bowel secondary to vasculitis without granuloma formation but otherwise consistent with GPA	Vasculitic rash, palmar erythema, splinter hemorrhages, severe abdominal pain with ischemic colitis requiring subtotal colectomy and ileostomy, postop pulmonary hemorrhage, AKI, thrombocytopenia, and ICU transfer	Steroids, cyclophosphamide	Postop complications with pulmonary hemorrhage, AKI, thrombocytopenia, wound infection, peritonitis, necrotic leak, sepsis, and death
Stuckey et al^15^ 45/M	Acute pancreatitis	ESR 96, AST 390, ALT 634, ALP 1,410, amylase 55, repeat amylase within 2 mo 239	CT abdomen: diffuse enlargement of pancreas with multiple small lesions of low attenuationRepeat CT abdomen: large pseudocyst	Biopsy (parotid gland): arteries showed marked angiocentric and angiodestructive vasculitis with occasional giant cell granulomata compatible with GPA	CXR shows multiple large pulmonary masses, CT chest shows more lesions and cavitation	Steroids, cyclophosphamide	CXR 2 months later showed only a small amount of residual disease, abdominal ultrasound was normal, ANCA was negative
Joshipura et al^16^ 47/M	Acute pancreatitis	ESR 49, amylase 874, lipase 1,294, positive c-ANCA with high anti-PR3 levels 156	Abdomen US: bulky body and tail of pancreas with altered echotexture CT abdomen: bulky and edematous pancreas	N/A	Marked improvement in arthralgia, nasal and ocular congestion, and abdominal pain	Steroids, cyclophosphamide	Clinical remission at 6-months follow-up
Chawla et al^17^ 60/F	Acute pancreatitis	Lipase 1,316, positive c-ANCA with high anti-PR3 levels 45.1	CT abdomen: diffusely edematous pancreas and a possible hypoattenuated lesion in the head of the pancreas with peripancreatic inflammatory changes EUS: 2 small heterogeneous hypoechoic lesions in the head and tail of pancreas and changes suggestive of acute pancreatitis	Renal biopsy: pauciimmune focal necrotizing glomerulonephritis	Pulmonary embolism, myocardial infarction, complete heart block, central diabetes insipidus, cavitary lung lesions	Steroids, cyclophosphamide, azathioprine (maintenance)	Clinical remission at 10-months follow-up
Matsubayashi et al^17^ 65/M	Acute pancreatitis	Trypsin 550, elastase-I 440, mildly high CA19-9, low amylase 34, positive c-ANCA and anti-PR3 levels 14	CT abdomen: enlargement of the whole pancreas	Autopsy: hemorrhagic pneumonia with giant cell granuloma, diffuse necrotizing pancreatitis, nephritis, and splenic granuloma	Vertigo, hearing loss, episcleritis, bloody sputum, arthralgia, hemorrhagic pneumonia, massive pulmonary effusion	No immunosuppressive therapy due to active infection	Hemorrhagic pneumonia leading to death
Valerieva et al^18^ 62/F	Acute pancreatitis and pancreatic mass	Normal ESR, CRP 15.8, normal amylase and lipase, positive c-ANCA and anti-PR3 levels 89.6	Abdomen US: slightly enlarged and hypoechoic pancreatic body and tail with blurry margins, as in acute pancreatitis CT abdomen: edema of the pancreatic tail without fluid collections or other abnormal findings MRI: 3 cm soft-tissue formation in the pancreas tail without pancreatic duct abnormalities	Biopsy (pancreas/spleen): typical vasculitis with fibrinoid necrosis, granulomas, and giant cells suggestive of GPA	Fever and severe abdominal pain despite antibiotics	Steroids, azathioprine	Clinical remission at 6-months follow-up

AKI, acute kidney injury; ALP, alkaline phosphatase; ALT, alanine transaminase; ANCA, antineutrophil cytoplasmic antibody; AST, aspartate transaminase; CRP, C-reactive protein; CT, computed tomography; CXR, chest x-ray; ERCP, endoscopic retrograde cholangiopancreatography; ESR, erythrocyte sedimentation; EUS, endoscopic ultrasound; GPA, granulomatosis with polyangiitis; Ig, immunoglobulin; LFT, liver function test; MPO, myeloperoxidase; MRCP, magnetic resonance cholangiopancreatography; MRI, magnetic resonance imaging; N/A, not available; US, ultrasound.

In summary, acute pancreatitis is a rare initial presentation of GPA vasculitis, and the diagnosis is often challenging in the absence of other organ involvement. Here, we illustrated a case of GPA presenting initially as acute pancreatitis, and it is quite atypical in its disease progression. As such, it is important to consider autoimmune diseases and systemic vasculitides in idiopathic pancreatitis after ruling out other common causes. A high clinical suspicion allows for early diagnosis and timely treatment with immunosuppressive therapy which achieves remission in 85%–90% of patients and increases survival.^11,12^ This knowledge is important for clinicians to keep a broad differential and consider vasculitides in clinical practice for prompt diagnosis and best patient outcomes.

## DISCLOSURES

Author contributions: M. Youssef and M. Sedarous wrote the manuscript and conducted the literature review. L. Hookey critically reviewed and approved of the article. A. Grin provided the pathology images. A. Chung provided the radiology images. All authors reviewed and approved of the whole manuscript. M. Youssef is the article guarantor.

Financial disclosure: None to report.

Informed consent was obtained for this case report.
